# Acceptability and feasibility of online occupational performance coaching for parents of children with disabilities in the UK

**DOI:** 10.1177/03080226251340508

**Published:** 2025-08-12

**Authors:** Tai Frater, Carolyn Dunford, Silvia Zumaglini, Fiona Graham, Dido Green

**Affiliations:** 1 Brunel University of London, Uxbridge, UK; 2 University at Buffalo, Buffalo, NY, USA; 3 University of Otago, Christchurch, New Zealand; 4 Jöonköping University, Jönköping, Sweden

**Keywords:** Occupational performance coaching, children, parents, participation, telehealth

## Abstract

**Introduction:**

Occupational performance coaching is a collaborative, strengths-based approach used by occupational therapists working with children and families. This study evaluates the acceptability and feasibility of delivering an online occupational performance coaching programme for parents of children with disabilities in the United Kingdom.

**Method:**

Mixed-methods feasibility study. Eight UK-based families with children with disabilities participated in four to eight online coaching sessions. The Canadian Occupational Performance Measure was used to measure changes in occupational performance. The Parenting Stress Index Fourth Edition (short form) was used to measure changes in parental stress. Six parents were interviewed, and four therapists participated in a focus group to explore their experiences of the programme.

**Results:**

Parents and therapists reported a high level of acceptability for the telehealth coaching intervention. Parents reported positive changes in occupational performance (mean difference 3.29; *p* = 0.01) occupational satisfaction (mean difference 3.99; *p* = 0.01). Parents’ average total stress decreased by 12.5 points with greatest reductions in the parental distress subscale. Therapist fidelity to intervention was relatively low.

**Conclusion:**

The coaching programme was acceptable for parents who responded well to the intervention with noted gains in occupational performance and satisfaction. Programme delivery was feasible for therapists though further training in occupational performance coaching is recommended.

## Introduction

Occupational therapists enable children to participate in activities they need, want or are expected to do in their everyday lives at home, school and in the community. There is growing understanding that to successfully support children’s participation occupational therapists must attend to the competence and capacity of parents (Grandisson et al., 2023; King et al., 2017; Novak and Honan, 2019). Parental well-being is also key given the increased likelihood of mental health conditions in parents of children with disabilities (Barreto et al., 2020; Scherer et al., 2019) and impact that parenting stress can have on child and family outcomes (Osborne et al., 2008).

Parent coaching is one approach used to support families of children with disabilities. Core components of parent coaching include a partnership where the coach listens and empathises with the family, parent-selected goals, collaborative analysis between the parent and coach, facilitation of parent’s reflective self-discovery and a focus on capacity building in the family (Graham et al., 2024a). Active involvement of parents in coaching is congruent with family-centred practice, where parents are engaged as active partners in their child’s therapy (King et al., 2017) and supports delivery of the National Health Service (NHS) Long Term Plan (2019).

The NHS Long Term Plan proposed a paradigm shift in the way NHS services, including children’s diagnostic and therapy services, are delivered (NHS, 2019). This involves moving more care into the community, increasing use of telehealth and digitally enabled care, a greater commitment to prevention, reducing waiting lists and supporting self-management. The NHS plan encompass the care provided to all United Kingdom (UK) children including the 11% of UK children estimated to have a disability, defined as a physical or mental health condition lasting over 12 months affecting daily activities (UK Parliament, 2023). It demonstrates a clear need for effective digital occupational therapy interventions for children and their families in theUnited Kingdom.

## Literature review

**Figure 1.**
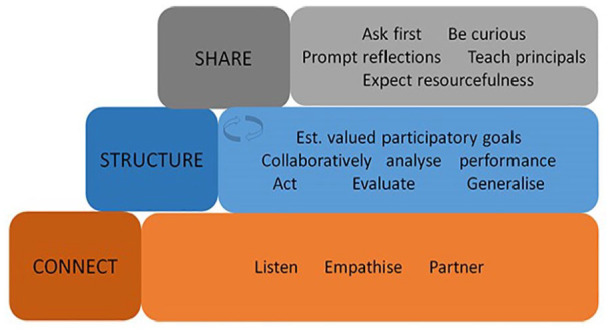
The three enabling domains of Occupational Performance Coaching. Reproduced with permission from Graham (2020). This work is licensed under a Creative Commons Attribution-NonCommercial-NoDerivatives 4.0 International License. University of Otago. Est: Establish.

Emerging evidence supports coaching parents of children with disabilities to improve a range of outcomes relevant to occupational therapists including occupational performance, motor, cognitive and educational outcomes (Graham et al., 2024a; Novak and Honan, 2019). Occupational Performance Coaching (OPC) is a coaching intervention that focuses on enhancing occupational performance in home, school and community contexts (Graham et al, 2021). OPC is framed as three enabling domains: Connect, Structure and Share that are hierarchically organised (Graham, 2020; Figure 1). A focal feature of OPC is the use of collaborative performance analysis to support achievement of ‘valued participatory’ goals. These are goals which detail an activity linked to a valued life role that are performed within a social context (Graham et al., 2021).

OPC has growing evidence to support improvement of children’s occupational performance and parental self-efficacy in families of children experiencing occupational challenges (Allen et al., 2021; Angelin et al., 2021; Graham et al., 2013), particularly: autistic children, children with cerebral palsy and children with developmental delay (Graham et al., 2024a). Evidence to date includes four randomised controlled trials (RCTs) (Ahmadi Kahjoogh et al., 2019; Bernie et al., 2023; Chien et al., 2024; Jamali et al., 2022) including a well-powered double-blind RCT which found OPC improved community participation which was maintained 8–9 weeks after the intervention (Chien et al., 2024). As an intervention that is delivered through a series of conversations OPC has excellent potential to be delivered via telehealth.

Telehealth interventions are those delivered remotely via videoconferencing or telecommunications technology. A recent systematic review found telerehabilitation to be equally, if not more effective than face-to-face delivery for children with disabilities (Ogourtsova et al., 2023). Understanding is growing on the conditions that support positive telehealth interactions with family choice, practitioner confidence and collaborative communication being cited as supportive conditions (Graham et al., 2023). OPC research indicates positive child and parental outcomes occur through telehealth delivery (Bernie et al., 2023; Chien et al., 2020, 2024; Jamali et al., 2022), with one small RCT demonstrating greater retention of parents receiving telehealth OPC compared with face-to-face delivery (Bernie et al., 2023). This is encouraging however more research is needed to further explore the relationships between contexts, mechanisms and outcomes in telehealth delivery of OPC.

OPC has been studied internationally with RCTs conducted in India (Angelin et al., 2021), Iran (Ahmadi Kahjoogh et al., 2019; Jamali et al., 2022), Hong Kong (Chien et al., 2024) and Australia (Bernie et al., 2023). OPC has not yet been explored in the United Kingdom in either face-to-face or telehealth delivery and differences in culture and healthcare systems may affect generalisability of existing study findings to a UK population. For example, whilst Australia and the UK are both Westernised, high-income countries as a country with a remote population Australia’s telehealth provision is historically more widely established, funded and promoted compared with that of the UK (Fisk et al., 2020). Despite more recent expansion telehealth approaches remain novel, and this could impact on local feasibility and acceptability to UK families and therapists.

The aim of this study was to explore the acceptability and feasibility of applying OPC via telehealth to parents based in the United Kingdom to inform future service delivery, and additionally consider potential benefits for children and their families. For the purposes of this study, we used Garizábalo-Dávila et al’s definitions whereby acceptability refers to ‘the perception of patients and health professionals versus determining whether the intervention is appropriate to address the problem, in reasonable, adequate, and convenient manner for its application in daily life’ and feasibility refers to ‘practicality of administering the treatment’ (2023: 6).

Specifically, our research questions were:

1. How acceptable is the coaching programme to parents and therapists in the United Kingdom?
2. How feasible is coaching programme delivery for parents and therapists in the United Kingdom?
3. What changes occurred in parent-reported occupational performance, occupational satisfaction and parenting stress levels?

### Method

The study design is a mixed-methods study incorporating a one group repeated measures pretest–post-test study and qualitative analyses of participant and therapist perspectives of the intervention and procedures. Qualitative methods, which can be used to inductively evaluate acceptability (Garizábalo-Dávila et al., 2023), were selected here to allow deep exploration of parent and therapist experiences. Quantitative and descriptive data were gathered on the fidelity to intervention and resources as proposed by Garizábalo-Dávila et al. (2023) as well as considering potential change in meaningful outcomes. Ethical approval was provided by the university research ethics committee.

### Participants

Families were recruited via promotions on Twitter and university webpages. Interested parents were sent study information and potential participants were then screened for eligibility via phone and provided written informed consent. Parents of children with a disability aged 0–18 living in the United Kingdom who could communicate in English and were able to access online sessions were included. Covid-19 restrictions in place in the United Kingdom at the time which influenced methodology and families were accepted without a formal diagnosis for their child due to limited diagnostic services.

### Data collection

1) Acceptability of the programme to parents and therapists in the United Kingdom.
  a) Qualitative interviews of parents following completion of programme.
  b) Therapist focus group following intervention.
2) Feasibility of the of the programme to parents and therapists in the United Kingdom.
  a) Resources required to implement the coaching programme:
    i) Record of training process
    ii) Case notes indicating time taken and number of sessions
  b) Fidelity analysis of videotaped sessions using the Occupational Performance Coaching Fidelity Measure (OPC-FM).
3) Changes in parent-reported occupational performance, occupational satisfaction and parenting stress levels:
  a) Canadian Occupational Performance Measure (COPM) performance and satisfaction scales
  b) Parenting Stress Index 4th edition Short-Form (PSI-4-SF)

### Measures

#### Occupational performance coaching fidelity measure (OPC-FM) (Graham et al, 2021)

The occupational performance coaching fidelity measure (OPC-FM) is an 18-item measure designed to evaluate whether OPC is being delivered as intended that can be self or expert rated. There are 14 items that focus on practitioner fidelity to OPC and four evaluating client response. Each item describes a behaviour that is rated on a four-point Likert scale from absent (0) to high consistency (3), with three items ‘distinguishing factors’ reverse scored. Three items are only scored on second or subsequent sessions. A total score is generated as a percentage of the maximum total score. The OPC-FM is in the process of validation as part of a larger study (Graham et al., 2024b).

#### Canadian Occupational Performance Measure

The Canadian Occupational Performance Measure (COPM) is an individualised outcome measure designed to assess self-perception of occupational performance and satisfaction that is valid, reliable and responsive (Law et al., 2005). The COPM is administered via a semi-structured interview between the therapist and parent to identify occupational performance issues which are then rated by the parents on a 10-point scale for importance, performance and satisfaction. Whilst not a goal setting measure, carrying out the COPM can support parents to identify goals for their children. The COPM is recommended for measuring progress in OPC (Graham et al., 2021).

#### Parenting Stress Index 4th edition Short-Form

The Parenting Stress Index 4th edition Short-Form (PSI-4-SF) (Abidin, 2012) was selected for this study to measure parental stress. It is widely used and has the best psychometric properties overall among the eight parenting stress measures studied by Holly et al (2019), demonstrating excellent internal consistency, validity and sensitivity. The PSI-4-SF is a 36 item self-report questionnaire that evaluates parenting stress levels. It is divided into three 12 item subscales: Parental Distress (PD), Parent-Child Dysfunctional Interaction (PCD-I) and Difficult Child (DC). It takes approximately ten minutes to complete, with parents rating each item on a five-point Likert scale. Higher scores indicate greater stress.

### Procedure

Parents completed the PSI-4-SF sent via post prior to their first session. The COPM was carried out within the first session with the occupational therapist with parents and goals set for the most important occupational performance issues identified (see Table 1). Coaching sessions were conducted via videoconferencing with minor exceptions of one session each for two parents which were carried out by phone due to technical issues on the day. Sessions involved collaboratively setting participatory goals, collaborative performance analysis, action planning and reflection on actions and progress towards goals following the procedures in the OPC manual (Graham et al., 2021). The COPM was re-administered by therapists in the final session and PSI-4-SF forms were sent to parents, with stamped self-addressed envelopes for return, following completion of the final session.

**Table 1.** Overview of goal areas by family.

Family	Number of goals	Goal subject	Occupational Performance areas	Example goal statement
1	3	Child	Sleep, feeding, social interaction/play	Son to try new flavours with minimal texture change onto crackers/crisps during snack time.
2	3	Child, mother	Self-care (morning and evening routine), leisure for Mum.	Son will complete his morning routine with no more than one prompt and a time prompt from parents without raised voices and push back in time to leave house at 8.10 am three days per week sustainably over a half-term.
3	4	Child, sibling, mother	Self-care (morning and evening routine), leisure (for Mum), school run	Mum will have 1.5 hrs time to herself downstairs watching TV with Dad most nights by mid-July.
4	1	Child	Handwriting	Son’s handwriting will be legible to everyone at home and school and to anyone that picks it up by end of year 4, and he will not be worrying about it.
5	2	Child	Self-care (dressing), cutting with scissors	For son to cut a page in half independently as part of a collage.
6	3	Child, family	Self-care, school engagement, play and social interactions	Child will be able to participate in a bike ride with parents twice a week.
7	1	Child	Self-care (dressing)	Child will independently dress herself with shoes and socks to leave for school calmly on 4 out of 5 days in a week.
8	2	Child	Self-care, play	Cleaning hands in water/flannel not on clothes and furniture/walls/stairs .

The coaching was provided by four experienced children’s occupational therapists who had completed an 8 hour online introductory course in OPC and self-directed study using the OPC Manual (Graham et al, 2021). Additionally, three of the therapists had carried out a 1 day in-person introductory course with the OPC creator. Therapists met fortnightly for peer support sessions and undertook monthly group mentorship sessions with an OPC endorsed trainer during the duration of the intervention period. Four videoed sessions from each therapist were randomly selected to be rated for fidelity using the OPC-FM by an expert rater with a mixture of initial and subsequent sessions.

Following the coaching programme parents were invited to take part in a semi-structured interview with a member of the research team based on their experiences. The interviewers were occupational therapists who had delivered the coaching programme but had not coached the parent interviewees. The interview schedule can be found in Appendix A. A focus group was facilitated by an external occupational therapist to capture the experiences of the four therapists who had coached parents. The focus group schedule can be found in Appendix B.

### Data analysis

*Question One*: Acceptability of the programme to parents and therapists in the UK

Parent interviews and the therapist focus group were separately analysed using six phases of thematic analysis proposed by Braun and Clarke (2022). Initially, familiarisation of the data was undertaken by watching and listening to video recordings and preparation of verbatim transcriptions, noting of initial ideas. Initial codes were generated through collecting and combining data with codes of relevant features. Themes were then generated by collating codes to possible themes prior to mapping themes and checking if codes and themes work with the extracted data. A final sixth step, involved analysis of extracts of data relating to the aims (Braun and Clarke, 2022). Quirkos software was used to support analyses, and an inductive approach was followed to allow themes to emerge from the data and reduce bias of any prior assumptions. Strategies for confirmability included investigator triangulation, reflective journaling and an audit trail.

*Question Two*: Feasibility of the programme to parents and therapists in theUnited Kingdom.
  Descriptive data analysis of session times was completed to indicate time commitment for both parents receiving the intervention and therapists delivering it. This involved calculating mean session times and ranges and median number of sessions. Descriptive analysis of fidelity scores was completed to explore how faithful to the intervention therapists were, given the training undertaken and mode of delivery. This involved calculating mean scores for initial and subsequent sessions, mean scores by therapist and number of sessions meeting 80% fidelity were calculated and ranges provided.
*Question Three*: Changes in parent-reported occupational performance and satisfaction and parenting stress levels.

Changes in COPM performance and satisfaction scores were reported and calculated as mean differences and changes pre- and post-values across goals, which varied in number between participants. Inferential significance was limited to non-parametric Friedman analyses to compare changes in goals and discussions limited to avoid misinterpretation. Descriptive data of PSI-4-SF mean differences and confidence intervals were analysed using IBM SPSS v28.0. Due to the exploratory nature of this study inferential statistics were minimally considered and limited to Wilcoxon matched pairs for pre- and post-comparisons of parent reported stress. In view of the fewer PSI-4-SFs returned mean differences and 95% confidence intervals (CIs) were provided and discussion limited to avoid misinterpretation.

## Results

### Participants

Eight families consisting of two mother–child dyads and one mother–father–child triad were recruited onto this study between April and July 2021. Parents recruited were aged between 35 and 53 with an average age of 43. Their children were aged between 2 years 1 month and 13 years 4 months and the most common diagnosis was autism (*n* = 5). All eight families completed the coaching programme. An overview of parent (*n* = 9) and child (*n* = 8) characteristics is provided in Table 2.

**Table 2.** Characteristics of family participants.

Parent Characteristics (*n* = 9)	
Parent gender (%)	
Female	8 (88.9)
Male	1 (11.1)
Parent mean age in years (SD)	42.7 (5.9)
Parent ethnicity (%)	
White (European and British)	7 (77.8)
Asian	1 (11.1)
Not specified	1 (11.1)
Parent educational level (%)	
Secondary	2 (22.2)
First degree	4 (44.4)
Higher degree	1 (11.1)
Not specified	2 (22.2)
Child characteristic (*n* = 8)	
Child’s gender (%)	
Male	6 (75)
Female	2 (25)
Child age in years	
Mean (SD)	9 (3.7)
Minimum-maximum	2-13
Siblings (%)	
No siblings	1 (12.5)
At least one sibling	6 (75)
Not specified	1 (12.5)
Child’s primary diagnosis (%)	
Autism^a^	5 (62.5)
Neurological condition	1 (12.5)
Prematurity and developmental delay	1 (12.5)
Awaiting formal diagnosis^b^	1 (12.5)

a One child with an additional diagnosis of anxiety

b One child awaiting assessment for developmental coordination disorder. Two children with other primary diagnoses were awaiting assessment for additional diagnosis of autism.

SD, standard deviation.

### Question 1: Acceptability of the programme to parents and therapists in the UK

#### Parent interviews

Six parents participated in interviews evaluating the programme acceptability. Qualitative analysis generated three acceptability related themes: (1) Step by step: A shared journey (2) Autonomy over my time and space and (3) Awakenings and insights leading to positive family changes.

##### Step by step: A shared journey

The first theme related to the perceived acceptability of the coaching intervention. Parents were positive overall ‘*a really lovely and positive experience*’ (P1). Goal setting helped define the destination and plan of how to get there: one parent who initially described the task ahead as ‘*a bit of a mountain’* explained ‘*I identified buttoning his shirts, tying his shoelaces and cutting using scissors. And then we worked on a plan out on how to get him engaged and making progress on those three areas*’ (P4).

Parents described breaking the journey into ‘*simple steps, small steps, but right direction’.* (P1). This supported a graded approach to strategies ‘*Rather than* “*Oh my God, he can’t do this. I’m thinking, well, we’re not worried about this bit. Let’s just go back a few steps and try and do this bit first. So looking at the in between steps*’ (P4). Progress reviews were helpful ‘*It was good just to realise actually with them that actually, wow, I’ve done a few things that more than I thought*’ (P6).

Therapists’ qualities were praised ‘*The person I worked with was warm, understanding. She was very reflective*’ (P2). Parent’s language reflected a strong partnership ‘*coming together and doing that problem solving. And solving together*’ (P4) which yielded results ‘*it was like I reached the goal by myself. But I know I didn’t do by myself*’ (P6). One parent wanted more direction from the therapist ‘*if you have a parent who just can’t find the way, maybe slightly deviate and give ideas*’ (P2).

##### Autonomy over my time and space

The second theme related to perceived acceptability of the mode of delivery. Parents valued the autonomy the programme offered over frequency, number, timing and duration of sessions which varied according to family preference. One parent described the programme as ‘*excellent in terms of accessibility and logistics*’ (P2). Parents unanimously valued the time telehealth delivery saved ‘*life is easier to fit in because it takes up less time*’ (P3) contrasting with in person appointments ‘*you end up losing half of the school day or half of your working day*’ (P4).

Parents liked seeing therapists faces from the comfort of their own home. For example, one parent who previously experienced anxiety attending external appointments, valued her ‘*calming*’ cat being on her lap and being able to share her space on her own terms. ‘*I get anxious when we have visitors around the house sort of tidying up and things. But you don’t need to do that on a Zoom call.’* (P3)

##### Awakenings and insights leading to positive family changes

The third theme relates to perceived acceptability of outcomes for the families. Parents were satisfied overall with progress ‘*We made a lot more progress during the coaching than when I was doing it on my own’* (P4). Parents also noted more global improvements in their children including improved confidence, fewer ‘meltdowns’ and better communication. ‘*It’s like a light bulb. Finally he’s able to say how he feels. It’s just a little small step. . . .So for me that’s a huge achievement.’* (P2)

Parents spoke at length about changes in their parenting approach ‘*It helped me to be more consistent in my approach with parenting and to try for longer with things before giving up’* (P3) ‘*recognising things which are doable or not doable for both myself and my child’* (P5). Expectation adjustment made parenting more rewarding ‘*giving you time and you enjoy small victories and so let you relax a bit’* (P1).

Overall outcomes were varied but all acceptable as summarised by one parent ‘*I hoped that the coaching would help our family life be a bit easier and it did make our family life easier.’* (P3)

#### Therapist focus group

All four therapists who delivered the intervention participated in a focus group to explore their experiences. Qualitative analysis of the therapist focus group generated two themes: (1) Telehealth benefits outweigh challenges and (2) Support in developing new skills.

##### Telehealth benefits outweigh challenges

I really felt the benefits outweighed them [technical issues] and the fact that you could have a conversation with them at their convenience in their own home. . .be more flexible with the timing, more flexible with the location, and you’re not putting so much burden on a parent to find a space having to, oh sorry like drive to find a parking space and all that stuff. (TA)

Therapists reflected that the benefits of online delivery outweighed the challenges they experienced as it strengthened rapport and technology challenges were minimal. The occupational therapists were surprised to note that online delivery strengthened rather than hindered therapeutic rapport.

I was extraordinarily surprised because I thought that we’d need to have at least one face-to-face to establish that relationship and I realised I didn’t need to do that. (TB)

They also found a window into the family’s home was particularly beneficial to provide a snapshot of living environments and family dynamics.

Being able to see them in their environment and that intimacy that comes from that and a sort of unique perspective of how their family dynamics work and things but, from, from my point of view it helped with the rapport building because they were seeing into my world as well. (TD)

But having experienced it [delivering OPC online], I see that actually it empowered families to because they were in control of the environment in which they were situated in and let and invited you into share. (TB)

Occupational therapists also noted that the flexibility and adaptability of online delivery offered significant advantages for both themselves, and the parents.

##### Support in developing new skills

The second theme pertained to therapists’ appreciating support from the mentor and peers in learning to deliver this intervention approach and delivery method. Their perceived inexperience resulted in anxiety whilst they were learning.

That whole being a novice again, so we’re all. . .so very experienced in our fields and then we’re being asked to deliver something that’s completely new to us so it’s that imposter’s syndrome of me going ‘oh yeah I totally know what I’m doing yeah, yeah it’s fine’. But. . .the anxiety was here, the expectations were it might be a nice chat, the reality was we sat there in an anxiety ridden ball and thinking am I doing the right thing, am I saying the right thing the right way, what’s going on? (TD)

Therapists identified the OPC mentor relationship and peer support from the other therapists as key facilitators for their learning and development.

She [the mentor] was very practical and just discussed very real examples from our own coaching sessions [from mentorship sessions], my one-to-one session was helpful as well. (TC)

I think if I was doing it on my own, I would have found it very stressful, but, but it was a very supportive and nurturing way to learn. (TA)

All those anxieties so having done it as a group was really important in the acquisition of the skill, and translation to practice. (TB)

### Question 2: Feasibility of the of the programme to parents and therapists in the UK

Parents participated between four and eight sessions of OPC with a median of seven sessions. Sessions were 26–73-minutes duration (mean 49 minutes), and the mean average total contact time was 321 minutes per parent. Therapists estimated associated record keeping and preparation took an additional 15–30 minutes per session.

#### Descriptive analysis of fidelity scores

The range of fidelity for initial goal setting sessions was 53%–93% with an average of 70%. The range of fidelity for subsequent sessions was 43%–81% with an average of 64%. Individual therapist averages varied from 56%–72% and the overall average was 67% fidelity. In total four sessions out of the twelve rated met the 80% proposed benchmark for fidelity (Graham et al., 2021). The item with the highest average fidelity was item 18: ‘*Therapist uses hands on techniques*.’ This item is reverse scored as hands on techniques are not congruent with OPC. The item with lowest average fidelity was item 10: ‘*Therapist prompts client generalising successful strategies to other valued activities, contexts and roles*.’ Full fidelity results are available as a Supplemental File.

### Question 3: Changes in parent-reported occupational performance, occupational satisfaction and parenting stress levels

**Figure 2.**
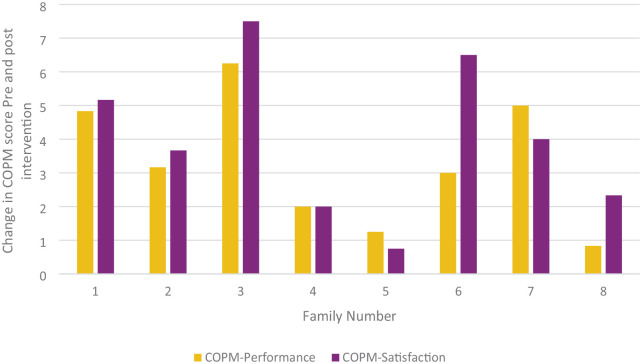
Mean COPM-Performance and COPM-Satisfaction change scores pre- and post-intervention by family. COPM: Canadian Occupational Performance Measure.

Data were available for eight families for the COPM. The mean COPM performance score post-intervention was significantly higher than pre-intervention (Mean difference 3.29; *z* = 2.52 *p* = 0.01; distribution χ^2^ = 8.00, *p* = 0.005). The mean COPM satisfaction score post-intervention was significantly higher than pre-intervention (Mean Difference 3.99; *z* = 2.52 *p* = 0.01; distribution *χ*^2^ = 8.00, *p* = 0.005). All COPM performance and satisfaction scores increased pre- to post-intervention and change scores can be seen in Figure 2.

Data were available for six families for the PSI-4-SF. Missing data from the PSI were managed by imputing the individual’s average of the relevant subscale as indicated in the PSI-4-SF manual (Abidin, 2012). For one participant three items of post data were imputed using last observation carried forward method, with the assumption of no change (Houck et al. 2004). Total stress scores post-intervention were lower than pre-intervention (Mean Difference −12.5; CI .194–24.81). All mean scores of subscales decreased with greatest decreases noted in the PD subscale (Table 3).

**Table 3.** Mean PSI-4-SF Scores pre- and post-intervention.

Variable	Pre-intervention		Post-intervention		Mean difference (Mdn difference)
	Mean (Mdn)	Range	Mean (Mdn)	Range	
PSI-4-SF Total (*n* = 6)	113.3 (113.5)	71–137	100.8 (100.0)	74−136	−12.5 (−13.5)
PSI-4-SF PD (*n* = 6)	37.0 (37.5)	23–51	31.5 (29.5)	23−42	−5.5 (−8.0)
PSI-4-SF PCDI (*n* = 6)	34.3 (36.0)	21–44	30.7 (31.5)	21−40	−3.7 (−4.5)
PSI-4-SF DC (*n* = 6)	42.0 (45.5)	22–52	38.7 (39.0)	25−54	−3.3 (−6.5)

Mdn: Median; PSI-4-SF: Parenting Stress Index 4th edition Short Form; PD: Parental Distress subscale; PCDI: Parent–Child Dysfunctional Interaction subscale and DC: Difficult Child subscale.

## Discussion and implications

This study explored the feasibility of delivering an online parent directed OPC intervention and considered the perspectives of parents and therapists as well as potential effectiveness.

Our first research question related to acceptability of the programme to parents and therapists in the United Kingdom. Parents’ responses indicate that the programme was positively perceived and deemed an acceptable intervention highlighting partnership working and collaborative problem-solving. This aligns with previous research that demonstrates consistent satisfaction with OPC Intervention (Angelin et al., 2021; Bernie et al., 2023) and which also emphasises positive therapist support and collaborative relationships (Ahmadi Kahjoogh et al., 2020; Chien et al., 2020). Themes highlighted in previous research that related to society or extended family (Angelin et al., 2021; Ahmadi Kahjoogh et al., 2020) were not repeated in this study, and this may be related to the different cultural contexts of the UK, India and Iran.

The pandemic acted as a catalyst for a shift to telehealth as a delivery method for therapies. In line with previous research (Bernie et al., 2023; Chien et al., 2020; Graham et al., 2023; Ogourtsova et al., 2023) parents and therapists in this study were positive about telehealth as a flexible and convenient approach that supports parent autonomy. The sample population were highly educated and, due to self-selection and online recruitment methods, are likely to be digitally literate and have access to Wi-Fi and hardware. It is not therefore possible to generalise their experiences to the UK population more broadly where digital exclusion remains a key concern (Office for National Statistics, 2019). It is recommended that therapists who are using telehealth for coaching are mindful of digital exclusion issues and adapt their approach accordingly including offering lower tech options when needed.

Our second research question related to feasibility of implementation. Session numbers were similar to those described in other studies albeit exceeding the four sessions of OPC combined provided by Bernie et al (2023). In the current study therapists demonstrated difficulty applying OPC to meet the fidelity criteria set for face-to-face sessions, with only a quarter of rated sessions meeting the 80% fidelity benchmark. Apart from Chien et al. (2020) the 80% benchmark has proved consistently challenging to meet in the literature (Bernie et al, 2023; Chien et al., 2024; Jamali et al., 2024) though this study’s fidelity results were lower than most. This is a limitation of the study and consequently findings should be interpreted with caution.

Possible explanations for low fidelity include expert rating rather than self-rating like Jamali et al. (2024), less-extensive training in OPC falling short of the 24 training hours recommended for research (Graham et al., 2021) and the fidelity measure needing adaptation for online delivery. Unusually, this study also included the initial sessions applying the COPM within our fidelity sample. Therapists in this study may have struggled to adapt the COPM, which assesses current occupational performance problems, to align with the OPC approach, which emphasises envisioning desired future states (Graham et al., 2021). For this reason, it may be preferable to conduct fidelity analysis of sessions once goals have been established, or to use goal attainment scaling (GAS; Turner-Stokes (2009), which focuses on desired goals and expected outcomes. In line with Chien et al. (2024), there was variety noted in individual therapist fidelity despite similar training and mentorship. Further training may improve quality of OPC delivery and reduce variability in fidelity as well as alleviating some of the therapists’ expressed anxieties about delivering the intervention. It may also complement the peer support and OPC mentorship that therapists found helpful. Further research on the impact of fidelity on outcomes is warranted to ensure understanding of the salient ingredients of the OPC approach in differing delivery contexts.

Our third research question related to potential changes in occupational performance, satisfaction with occupational performance and parenting stress levels. The significant improvements found in occupational performance and satisfaction is encouraging. These results align closely with previous coaching studies examining effectiveness (Angelin et al., 2021; Bernie et al., 2023; Chien et al., 2020, 2024; Graham et al., 2018; Graham et al, 2021; Jamali et al, 2022). Considering the design of the study these results should be viewed with caution. With small numbers and no control group, it is hard to discount the role of influencing factors, including the pandemic context as highlighted by the parents who expressed their gratitude for help at this challenging time. It is also of note that the COPM was administered by the therapists who completed the coaching intervention with the parent, and this could have biased the results. This was an intentional design choice to support goal setting and rapport building with the intervention therapist; however, other OPC studies have blinded assessors (Ahmadi Kahjoogh et al., 2019; Bernie et al., 2023; Jamali et al., 2022) or both assessors and participants (Chien et al., 2024), and this is an important design consideration for future studies.

The reduction in parenting stress is also encouraging given that high levels of parental stress may moderate the effectiveness of interventions on child outcomes (Osborne et al., 2018). Three other intervention studies have examined stress as a secondary outcome. While Chien et al. (2020, 2024) did not find significant results, Bernie et al (2023), who also used the PSI-4-SF, identified a 14.5 percentile decrease in the total score for the telehealth arm in contrast to small increases in stress in the usual care and face to face arms. It should be noted however that the numbers presented in Bernie et al.’s (2023) study and here are too low for firm conclusions to be drawn. Additionally, there were some implementation issues with the PSI-4-SF in this study with low return rates, missing data and concerns raised by two parents with the wording of one of the items in the ‘Difficult Child’ subscale. The validity of this measure for parents of autistic children has been explored previously by Zaidman-Zeit et al. (2010) who proposed that the ‘Difficult Child’ and ‘Parent–Child Dysfunctional Interaction’ subscales may not be valid for this population. It is proposed that other measures of parenting stress including those designed for autistic populations would be worthy of consideration for future programmes. Additionally, online or therapist administration may reduce missing data due to the added time and administrative burden of postal returns.

## Conclusion

This study sought to evaluate the acceptability and feasibility of an online coaching intervention based on OPC delivered to UK-based parents during the pandemic. The intervention, including the telehealth delivery, was found to be broadly acceptable for parents and therapists. Parents achieved notable gains in occupational performance and satisfaction and a reduction in parent stress; however, these results should be interpreted with caution given the study design. Further areas that require exploration relate to therapist training and measurement of fidelity with telehealth delivery, and appropriateness of outcome measures such as those measuring parental stress. Overall, this intervention demonstrates good potential to benefit UK-based parents via telehealth.

### Key findings

- The OPC telehealth intervention was acceptable and feasible for families and therapists.
- Family occupational performance improved and parental stress reduced following OPC.
- Fidelity to intervention was low and further OPC training may support consistency of higher-quality OPC delivery.

### What this study has added

- This study adds to the growing evidence base supporting occupational performance coaching as an acceptable and feasible intervention to be delivered online for parents of children with disabilities.

## Supplemental Material

sj-docx-1-bjo-10.1177_03080226251340508 – Supplemental material for Acceptability and feasibility of online occupational performance coaching for parents of children with disabilities in the UK Supplemental material, sj-docx-1-bjo-10.1177_03080226251340508 for Acceptability and feasibility of online occupational performance coaching for parents of children with disabilities in the UK by Tai Frater, Carolyn Dunford, Silvia Zumaglini, Fiona Graham and Dido Green in British Journal of Occupational Therapy

## Appendix A

### Semi-structured interview guide–parent participants

Experience of OPC coaching programme

- How did you find the coaching programme?
  - What were your expectations ahead of the coaching programme?
  - Did the coaching programme meet these expectations?
  - Previous experiences

Experience of online delivery

- Was there anything about this programme that particularly helped meet your goals for your child?
  - Did they achieve these goals?
  - Experiences with the platform
  - Relationship with OT
  - Accessibility
- Was there anything about this programme that prevented meeting your goals for your child?
  - Were any goals not achieved?
  - Technology/technical issues
  - Time/Duration
  - Agenda that was set
- Can you identify any support requirements needed during the programme?
  - Relationship with OT
  - Webcam
  - Space for online intervention
  - Any logistical aspects
  - Any issues with managing other children/pets/work while taking part in the programme?
  - Scheduling aspects

Impact on Parenting roles

- Did the OPC programme change your thinking or behaviour?
  ■ If so, in what way?
  ■ Any changes in yourself? E.g. Knowledge/motivation
  ■ Any changes in your child?
    • Have you noticed any changes in your confidence managing your child’s tasks?
  ■ Do you think that the OPC programme changed the way you support your child? If so, in what way?
  ■ Have you noticed any changes in the relationship you have with your child?
  ■ Changes in other family relationships?

Implications for Future Research

- What changes if any would you suggest to us?
  - Technology-related changes
  - Frequency/Duration of sessions changes

## Appendix B

### Focus group topic guide – therapist participants

Experience of learning to deliver the OPC approach through an online medium

1. How did you find the materials and resources that were provided for learning the approach
2. How did you find the supervision/mentoring experience?
  - What were your expectations ahead of your continuing professional development learning experiences
  - From your perspective methods and procedures for learning meet y our needs?
  - Previous experiences in post-graduate professional training

Experience of delivering the OPC coaching programme

- How did you find delivering the coaching programme?
  - What were your expectations ahead of your participation in the coaching programme?
  - From your perspective as a clinician, did the coaching programme meet these expectations?
  - Previous experiences and comparisons with coaching or other interventions delivered online compared to face to face

Online delivery of the OPC coaching programme

- What are your thoughts regarding the online delivery: eg. format/technology/accessibility
- Duration/frequency/accessibility therapist

Processes and structures

- Impact of pandemic vs applications after the pandemic
- Useful approach/format for pandemic any differences

Future impact – Needs

- Influence on practice
- Influence on training mentorship, peer support, management support needs
- Barriers
- Facilitators
