# Supplementary material for: Acceptability and feasibility of online occupational performance coaching for parents of children with disabilities in the UK
Source: Br J Occup Ther. 2025 Aug 12;88(9):563–73. doi: 10.1177/03080226251340508 (PMC12399807; doi:10.1177/03080226251340508)
Supplement: sj-docx-1-bjo-10.1177_03080226251340508 – Supplemental material for Acceptability and feasibility of online occupational performance coaching for parents of children with disabilities in the UK [file sj-docx-1-bjo-10.1177_03080226251340508.docx]

**Supplementary Materials. Expert rating of therapist fidelity to OPC using the OPC-FM**

|  | Item Descriptor | Therapist | | | | Mean |
| --- | --- | --- | --- | --- | --- | --- |
|  |  | A | B | C | D |  |
| Critical Components | 1. Therapist expresses empathy through comment & gesture, comprising non-judgmental responsiveness to the client’s emotional experience. | 2.5 | 2.75 | 2.75 | 3 | 2.75^a^ |
|  | 2. Therapist prompts client-led goal setting around a situation that is clearly highly meaningful to client. | 2.75 | 2.75 | 2.5 | 3 | 2.75^a^ |
|  | 3. Therapist prompts occupation/participation focused (activity + context) expression of the goal. | 2 | 3 | 3 | 2.75 | 2.69 |
|  | 4. The therapist prompts the client to envision the preferred, future goal situation. In subsequent sessions, the therapist refers to or prompts further clarification of the previously discussed vision | 1 | 1.75 | 0.75 | 2 | 1.38^b^ |
|  | 5. Performance analysis is oriented mostly to the preferred (goal) situation and solutions leading to it. (i.e., performance analysis is not oriented to the problem or current situation) | 1.5 | 2 | 1.75 | 1.5 | 1.69 |
|  | 6. Therapist prompts client-led performance analysis of the goal situation. Therapist prompts relate to client’s perceptions and understanding of goal situations rather than therapist understanding or perceptions. | 1.5 | 1.75 | 1.5 | 1.5 | 1.56 |
|  | 7. Therapist prompts client decision-making/choices about identifying and selecting solutions/strategies leading to goal achievement. | 1.25 | 2.25 | 1.75 | 2 | 1.81 |
|  | 8. Therapist prompts client to specify details of their action plan (i.e., when, where, how, with whom). | 1.25 | 1.5 | 2 | 1.25 | 1.5 |
|  | 9. Therapist prompts client evaluation of planned strategies and outcomes after they are attempted. (scored subsequent sessions only). | 0.5 | 1.67 | 0.5 | 1 | 0.92^b^ |
|  | 10. Therapist prompts client generalising successful strategies to other valued activities, contexts & roles (scored subsequent sessions only). | 0 | 1 | 0 | 1 | 0.5^b^ |
| Client Response | 11. Client seems to trust the therapist. | 3 | 2.25 | 2.25 | 2.75 | 2.56 |
|  | 12. Client articulates specific reflection and analysis of goal-related situations | 1.5 | 1.75 | 2 | 1.5 | 1.69 |
|  | 13. Client articulates specific planned actions within goal-related activities outside of direct contact with therapist. | 1.5 | 1.75 | 2 | 1.33 | 1.65 |
|  | 14. Client reports enacting actions intended to influence goal progress (including planned actions and innovations) in subsequent sessions (scored subsequent sessions only). | 1.5 | 1.67 | 2 | 1 | 1.54 |
| Distinguishing** Factors | 15. Therapist provides advice without implicit or stated permission. | 1 | 3 | 1.75 | 2.75 | 2.13 |
|  | 16. Therapist attempts to persuade client to agree with therapist’s interpretation or ideas. | 1.5 | 3 | 2.5 | 3 | 2.5 |
|  | 17. Therapist summarises or paraphrases the client’s words in their own words, rather than using client’s words | 1.25 | 2 | 1.75 | 3 | 2 |
|  | 18. Therapist uses hands on techniques (e.g., hand over hand) on the goal subject for the purposes of directly improving performance (excluding teaching or demonstrating a strategy to the client). | 3 | 3 | 3 | 3 | 3^a^ |
|  | Mean fidelity score (%) | 56 | 72.13 | 66.11 | 72.22 | 66.5 |

^a^ Three highest scoring items, ^b^ Three lowest scoring items

*Note. Critical components items 1-10 reflect the behaviours of the therapist, Client Response items 11-14 reflect the client’s response and Distinguishing factors item 15-18 are behaviours that are not congruent with OPC. Reverse scores are presented here for ease of comparison between items.*
